# Tomographic *in vivo* microscopy for the study of lung physiology at the alveolar level

**DOI:** 10.1038/s41598-017-12886-3

**Published:** 2017-10-02

**Authors:** Goran Lovric, Rajmund Mokso, Filippo Arcadu, Ioannis Vogiatzis Oikonomidis, Johannes C. Schittny, Matthias Roth-Kleiner, Marco Stampanoni

**Affiliations:** 1 Swiss Light Source, Paul Scherrer Institute, 5232 Villigen, Switzerland; 2 Institute for Biomedical Engineering, ETH Zurich, 8092 Zurich, Switzerland; 3 Max IV Laboratory, Lund University, SE-221 00 Lund, Sweden; 4 Institute of Anatomy, University of Bern, 3012 Bern, Switzerland; 5 Clinic of Neonatology, University Hospital of Lausanne (CHUV), 1011 Lausanne, Switzerland; 6 Present Address: Centre d’Imagerie BioMédicale, École Polytechnique Fédérale de Lausanne, Lausanne, 1015 Switzerland

## Abstract

Lungs represent the essential part of the mammalian respiratory system, which is reflected in the fact that lung failure still is one of the leading causes of morbidity and mortality worldwide. Establishing the connection between macroscopic observations of inspiration and expiration and the processes taking place at the microscopic scale remains crucial to understand fundamental physiological and pathological processes. Here we demonstrate for the first time *in vivo* synchrotron-based tomographic imaging of lungs with pixel sizes down to a micrometer, enabling first insights into high-resolution lung structure. We report the methodological ability to study lung inflation patterns at the alveolar scale and its potential in resolving still open questions in lung physiology. As a first application, we identified heterogeneous distension patterns at the alveolar level and assessed first comparisons of lungs between the *in vivo* and immediate post mortem states.

## Introduction

Most of the nowadays-available knowledge about the mammalian respiratory system, regarding both developmental and pathologic states, comes from animal studies^1^. Due to their similarities, rodents like rats or mice are commonly used as animal models^2^ filling the preclinical gap to diagnostic thoracic imaging in humans where up to date X-ray CT is still the most sensitive technique, despite being unable to resolve microscopic lung structures such as alveoli^3^. Although a vast amount of lung diseases affects these microstructures, *in vivo* studies of animals are nowadays mainly performed with micro-CTs and at low resolutions, either by prospective^4^ or retrospective^5^ respiratory gating. Typical pixel sizes in the order of 100 × 100 μm^2^ and dose rates in the range of 250 mGy to approximately 2 Gy for full 4D-datasets^6^ appear unsuitable for establishing further and deeper knowledge in pathogeneses.

Studies that require spatial resolutions toward the micrometer scale are therefore increasingly performed at third-generation synchrotrons, which provide high sensitivity to soft tissue by highly coherent X-rays and sufficient photon flux. Low-dynamic *in vivo* studies stemming from bone microarchitecture^7^, microvasculature^8^ and brain tumors^9^ as well as high-dynamic ones in the field of respiratory physiology in insects^10^ have been reported with remarkable results. More recently, the course of embryonic development in Xenopus laevis^11^ has been studied with synchrotron-based microtomography and an analogous technique called “projection-guided gating” has enabled the full deciphering of the 150-Hz blowfly flight motor in 4D^12,13^. However, all aforementioned studies have in common the fact that they were performed with much simpler animal models and experimental setups, compared to the case of *in vivo* lung studies in rodents.

At synchrotrons, so-called K-edge subtraction (KES) imaging^14^ with pixel sizes down to 50 × 50 μm^2^ and total radiation doses of up to 10 Gy has been applied to measure in 3D absolute regional lung volumes^15^, bronchial kinetics^16^ and airway function^17^ in rabbit animal models. Later on, propagation-based phase-contrast imaging was utilized to dynamically identify and locate airway liquid clearance in newborn rabbit pups^18^ and the application of single-shot phase retrieval^19^ allowed for quantifying the change of lung fluid. It is noteworthy that the subsequent quantitative functional and anatomical imaging of lung ventilation, which could now be performed at even smaller pixel sizes (about 20 × 20 μm^2^) and lower dose rates, directly influenced clinical practice in the treatment of preterm infants by exemplifying the beneficial aspects of positive end-expiratory pressure (PEEP)^20–22^. Subsequently, the original technique has been combined with particle image velocimetry (PIV) to study regional 3D displacement maps, but no advances were made in terms of spatial resolution^23,24^. Slight improvements were achieved by 4D lung imaging with a 11.8 × 11.8 μm^2^ pixel size and under quasi-static inflation, but the authors concluded that the technique was unable to resolve alveolar kinematics^25,26^. Finally, with the introduction of so-called tracking X-ray microscopy^27^ it became possible to track single alveoli during inspiration and expiration, although being limited to single alveolar ducts or regions of interest located close to an edge of the lung. None of the above methods were able to shed light to the questions of how lungs inflate and deflate at microscopic levels^28–30^.

To overcome previous challenges in high-resolution *in vivo* lung imaging we have developed a prospective heartbeat-gated synchrotron-based microtomographic technique. It allows studying lung inflation and deflation patterns at the alveolar scale, which to the best of our knowledge represents an improvement in spatial resolution by at least a factor of four compared to all previous studies. As a first application, we demonstrated the study of high-resolution inflation patterns at pixel sizes down to 1.1 × 1.1 μm^2^ and described for the first time the immediate microscopic *post mortem* effects on lung tissue by comparing selected volumes acquired first *in vivo* and then *post mortem*.

## Results

In the following, first the results affiliated with this new method itself are presented. Afterwards, its technical validation and performance are presented and finally results from first applications are described.

### Prospective heartbeat-gated imaging

**Figure 1:**
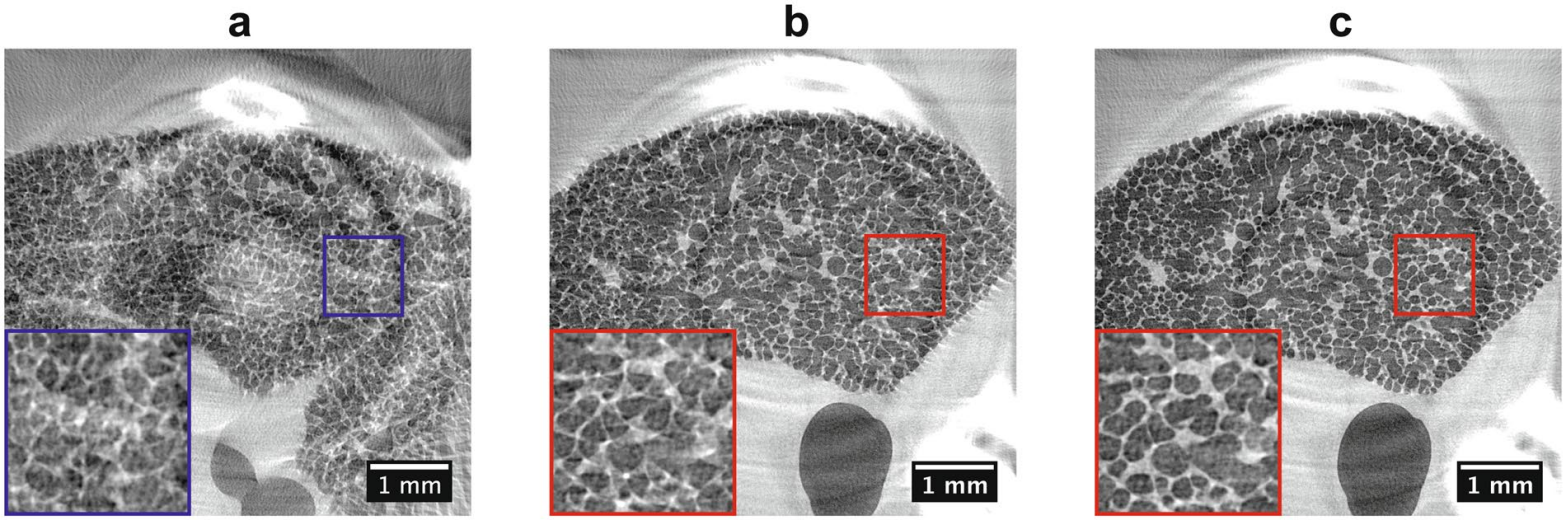
Influence of the timing of the single-projection exposure within the cardiac cycle as well as the correction for irregular heartbeat periods during a breath-hold phase under continuous rotation. Tomographic slices with approximately 450 angular projections, 2.9 × 2.9 μm^2^ pixel size, and 3 ms single-projection exposures are shown when projections are acquired at a 50 ms delay (**a**) and at a 150 ms delay (**b**) from the R-wave. Finally, since the angles of the tomographic projections are not sampled in an equiangular way, an artifact-free CT reconstruction of the same dataset (**b**) is obtained only after utilizing the tomographic angle of each projection in the CT reconstruction step (**c**).

We have developed an animal preparation, anesthetic and handling protocol combined with a prospective heartbeat-triggered gating technique for the tomographic *in vivo* imaging of lungs at the level of single alveoli and at dose rates permitting acute (terminal) experiments. At the microscopic level, the heart contraction has a significant influence on the lung motion. The fact that cardiac gating becomes necessary has already been investigated^25^, but it has only been established recently that ultra-short single-projection exposure times in the order of a few milliseconds are required as well^31^. In the present study, we have found that the most crucial setting in tomographic *in vivo* imaging of lungs at the level of alveoli is the time point within the cardiac cycle at which the imaging takes place. This setting requires keeping a record of the projection angles (Fig. 1), while performing a constant and continuous rotation of the sample, to account for irregular heartbeat periods and hence the non-equidistant angular sampling (as detailed in the Methods section). The recorded projection angles are then utilized during tomographic reconstruction.

Several experimental aspects must complement these results. The electrocardiogram (ECG) device typically senses the electric impulse just before the heartbeat by detecting the corresponding QRS-complex (or more precisely the R-wave). Each QRS-complex was then derived to create a trigger-signal that is routed to the acquisition hardware such as the camera and the X-ray shutter. In the most ideal case, the shutter and camera will act instantaneously and imaging will be performed at the time point when the R-wave occurs. However, there is an inherent minimal time delay between the QRS-trigger and the time point when the X-ray shutter is opened. As the QRS-complex is physiologically followed by the systolic contraction of the ventricles, this would mean that imaging would fall into the time span of strong cardiac movement, inducing motion of the neighboring lung tissue (Fig. 1a). To overcome this problem, our strategy was to adjust the delay between the QRS-complex and the shutter opening so that imaging would be performed shortly before the following R-wave, meaning at the end of the diastolic ventricular filling when there is little ventricular motion activity. Additionally, a so-called blanking time was set in order to ignore spontaneous heartbeats that would occur earlier than the required delay time of the acquisition hardware. Only the coupling of all these aspects and the fact that ultra-short single-exposure times of a few milliseconds were employed finally led to motion-less CT reconstructions (Fig. 1c).

### Technical validation

**Figure 2:**
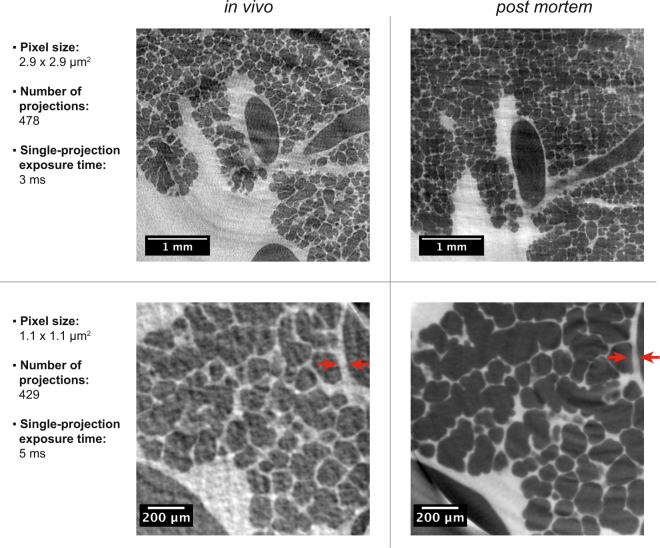
Comparison of the image quality of *in vivo* and *post mortem* tomographic slices of lungs at two different magnifications. The red arrows exemplify the difference in lung tissue thickness, which was observed between the *in vivo* and *post mortem* case. The *post mortem* tomographic slice was acquired at 30 cmH_2_O, while all other images were acquired at 15 cmH_2_O.

To fully evaluate the performance of the proposed imaging technique, we compared tomographic slices acquired with two different optics with pixel sizes of 2.9 × 2.9 μm^2^ and 1.1 × 1.1 μm^2^, respectively. With these two settings the scanned partial volumes were 5.8 × 5.8 × 2.7 mm^3^ and 2.2 × 2.2 × 2.2 mm^3^ in size, respectively. Tomographic acquisitions of selected lung regions were acquired first *in vivo* in anesthetized animals and shortly thereafter *post mortem*, both of them under constant breath-holds induced by the ventilator. As detailed in the Methods section, each *post mortem* scan was achieved after administering an overdose of pentobarbital to the anesthetized animal at the end of an *in vivo* scan. The direct comparisons (Fig. 2) yielded somewhat differing results between the two optics (magnifications). For the 2.9 μm-pixel-size optics the obtained image quality for both the *in vivo* and *post mortem* samples produced comparable results at a single-projection exposure time of 3 ms. For the 1.1 μm-pixel-size optics, however, the difference was quite significant due to the presence of heart-induced motion artifacts that were present in the lung during the *in vivo* scans. It shall be noted that for the 1.1 μm-pixel-size optics, due to the higher magnification (hence lower photon efficiency), a higher single-projection exposure time of 5 ms had to be used in order to produce comparable signal-to-noise ratios as in the case of the 2.9 μm-pixel-size optics.

**Figure 3:**
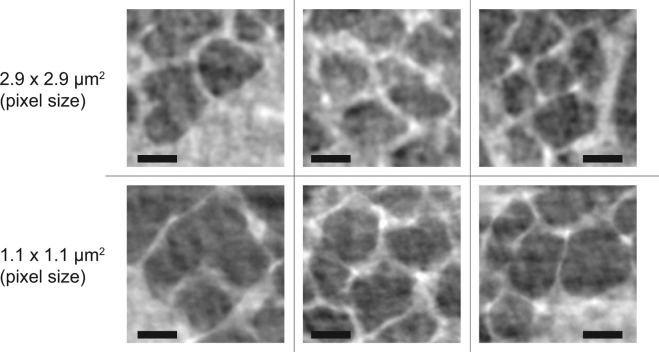
The six panels show selected small alveolar regions of *in vivo* tomographic slices from several experiments to compare the quality of images from the two different optics. The bright white points represent the triple points where septa meet. Additionally, imaging artifacts are visible as small speckles (dark and bright areas) within the alveolar airspaces. These artifacts originate from the low number of tomographic projections in combination with low single-projection exposure times. The comparisons between the two pixel sizes show that in the *in vivo* case the 1.1 μm-pixel-size optics bears no additional information in terms of improved detail resolution or signal-to-noise ratios. The scale bar represents 100 µm.

From these results, we concluded that there was no appreciable improvement by using the higher magnification for the *in vivo* scanning mode. To further test this hypothesis, we randomly selected several alveolar regions in images acquired with both of the magnifications to qualitatively investigate their informative content in terms of visible biological features. There was indeed no improvement (Fig. 3), neither in signal-to-noise ratio nor in spatial resolution, when the higher magnifying optics were used. This result is significant in the sense that it appears to be directly linked to the required temporal resolution of the imaging systems. Namely, it indicates that even shorter X-ray exposure times than the ones that were applied here (5 ms) would be necessary and to be combined with accurate triggering within the cardiac cycle. To achieve this under the current imaging settings would necessitate a significant increase (4–5x) of the X-ray photon flux.

In this respect it is important to briefly discuss the effects of radiation dose on lungs. Namely, as we report later on, the total absorbed dose for multiple scans of the same animal roughly added up to 90 Gy. Such high radiation doses are usually applied in radiation therapy where they cause imminent damages to lung tissue^32^. These damages can be assessed in different ways such as perfusion studies^33^, by measuring inspiratory rates^34^ or by histological examinations^35^, however, they usually occur in the order of weeks after irradiation. In the case of a 90 Gy irradiation through a 3 mm collimator, which directly compares to the fields of view present in our experiments, no significant morphological abnormalities (except mild interstitial inflammatory cell infiltration) were observed after one week^36^. These results roughly state the time window under which acute (terminal) *in vivo* experiments can be performed, where no immediate effects from the radiation on the investigated biological samples will be present. However, immediate radiation damage effects in biological soft tissue have been reported at localized doses in the order of 1.5 kGy^37^.

**Figure 4:**
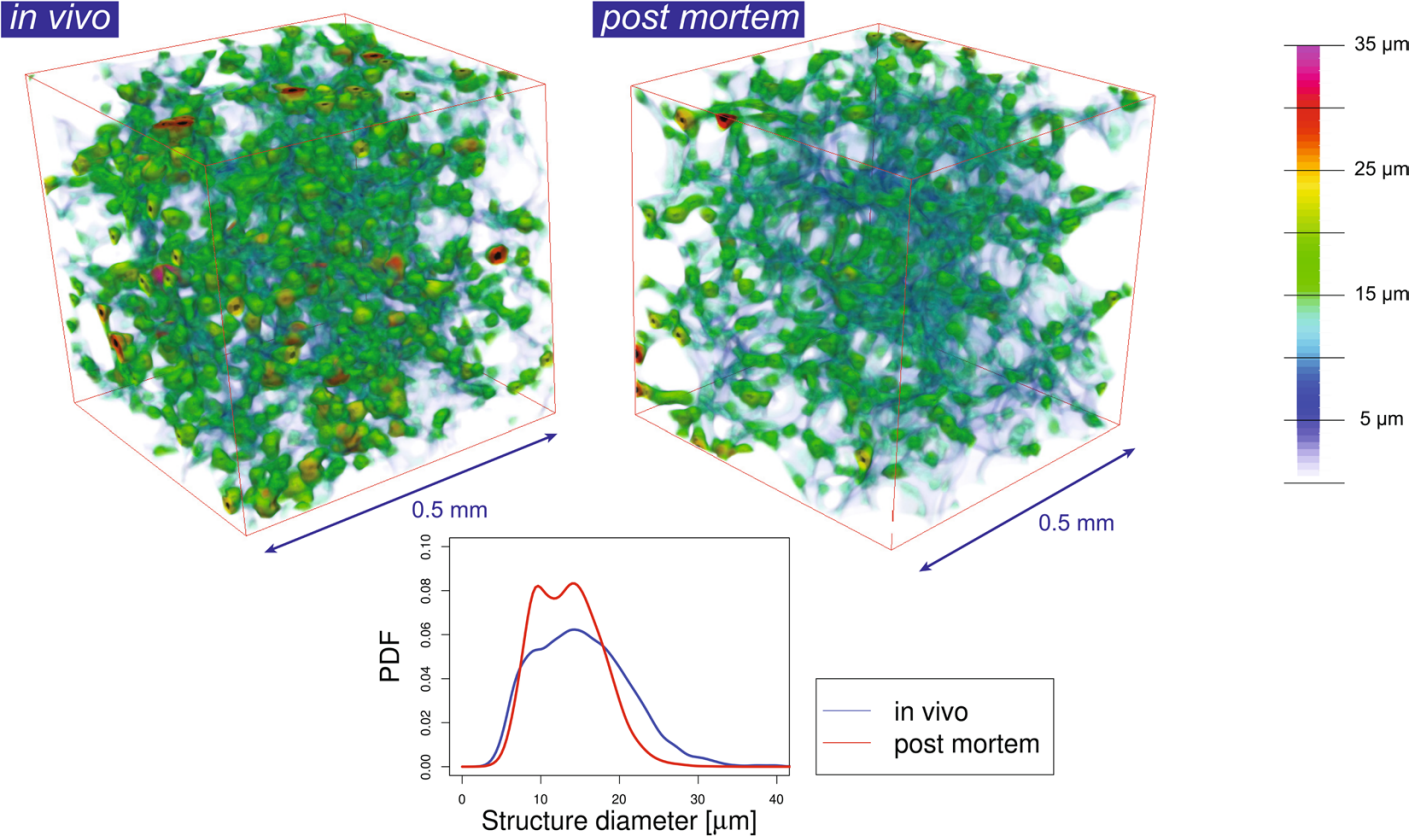
Comparison of lung tissue difference *in vivo* and the immediate *post mortem state*. 3D tomographic data that was obtained with the 1.1 μm-pixel-size optics was 3D median-filtered with a kernel of 7 pixels to remove noise artifacts and thus neglect structures below 9 µm. Following that, a thickness map analysis^38^ was conducted on the segmented 3D lung tissue data. As can be seen from both the 3D renderings and the thickness map probability density (PDF) plot, a significant decrease in lung tissue thickness is observable in the *post mortem* case that mainly affects structural diameters between 20–30 µm.

Several other aspects were found during these experiments that we note hereinafter. A comparison of the *in vivo* tomographic slices with the *post mortem* ones (Fig. 2) shows that the alveolar microstructures in the most cases can be well matched with each other. However, a fast-emerging stiffness increase of the lung was observed after the immediate *post mortem* state as well as a significant decrease in lung tissue thickness in the *post mortem* case. The former one usually can be measured directly with the ventilator, where an increase in air resistance appears shortly after *post mortem*. It is also slightly visible in Fig. 2 with the 2.9 × 2.9 μm^2^ pixel size where the *in vivo* image was inflated at 15 cmH_2_O and the *post mortem* image was inflated at 35 cmH_2_O pressure in order to obtain a comparable level of inflation. This observation, however, might also be associated with reaching the total lung capacity (TLC) and thus be directly linked to the hysteresis curve of the lung. On the other hand, the change in tissue thickness is indicated by the red arrows for the 1.1 × 1.1 μm^2^ pixel size (Fig. 2), which show areas where the tissue (alveolar septa including the blood vessels and surfactant) shows significant differences. This was also observed in 3D and by a quantitative thickness map analysis of the lung tissue^38^ (Fig. 4). Furthermore, in nearly all cases when comparing the *in vivo* to the *post mortem* data we observed a shrinkage of the lung tissue, meaning that features that were observed *post mortem* appeared thinner than in the *in vivo* scans. Hence, a one-to-one volumetric match was impossible. These findings might play a significant role as morphometric lung studies are often performed on freshly sacrificed animals and/or lung fixed samples^39^. The observed difference in lung tissue thickness cannot be unambiguously accounted for and might be explained by a halted blood circulation, inflammation and starting degradation processes in the *post mortem* state. We found that especially the larger structures (20–30 µm) are affected (Fig. 4). The here for the first time highlighted differences between *in vivo* and *post mortem* imaging demonstrate the challenge of making conclusions about how alveolar structures behave *in vivo* out of *post mortem* ventilation studies.

### Application to the study of different grades of pulmonary inflation

**Figure 5:**
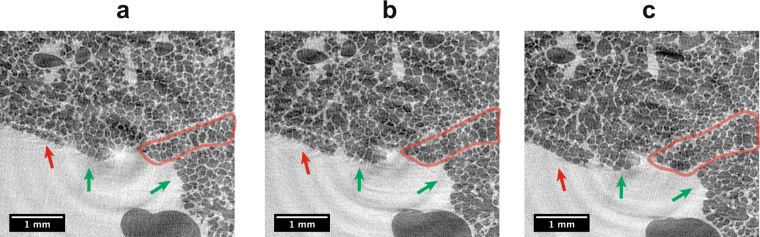
Lungs different grades of inflation at three different pressures: (**a**) 5 cmH_2_O, (**b**) 10 cmH_2_O and (**c**) 15 cmH_2_O. The green arrows mark matching lung structures while the orange line encircles a neighboring lobe that exhibits a stronger volume increase compared to the surrounding structures. The red arrows depict areas where different alveolar structures are being “pushed” into the field of view from above or underneath lying structures.

We applied our imaging method to study lungs different grades of inflation at the micrometer scale. We analyzed whole tomographic slices of selected lung regions under quasi-static inflation with the 2.9 μm-pixel-size optics (Fig. 5) and at three different inflation pressures: 5, 10 and 15 cmH_2_O. The green arrows in Fig. 5 indicate regions that can be matched unambiguously at different pressures, while the red ones depict areas where different structures are being “pushed” into the inspected region that were previously located below or above within the volume, with respect to the regarded tomographic slice.

By further analyzing the lung volume along the sagittal and transversal axis, we observed a strongly heterogeneous inter-lobar and intra-lobar distension pattern. As indicated by the orange marked area in Fig. 5, some regions exhibited stronger inflation compared to other areas in which alveolar structures did not increase significantly in volume. This indicated that the lung structures appeared to be ventilated by varying degrees. As a consequence, it was impossible to match all lung regions within one tomographic slice since the alveolar structures stretched differently within the respective lung region and by different amounts, depending on the spatial direction. Thus, during inflation the alveolar structures showed a more or less heterogeneous up-scaling, meaning that all alveolar structures increased their volumes - some more and some less - despite small deviations in their structural shape. Similar behavior was observed in all inspected regions of the investigated animals.

Due to data acquisition under quasi-static inflation, i.e. under a breath-hold phase, our conclusions are not necessarily valid for the real dynamic situation of a free-breathing or mechanically ventilated animal. Further, the primary aim was to decouple the cardiac motion from lung imaging, which is why a breath-hold approach was chosen. Our approach can be extended to different scenarios, e.g. by combining it with a slow inflation, in order to test the presence of disease-induced lung dynamics deteriorations. This, however, was beyond the scope.

## Discussion

Our proposed prospective heartbeat-gated synchrotron-based micro-CT renders it possible to study lung inflation patterns during diastole at the alveolar scale *in vivo*. The method appears suitable to study high-resolution lung structures either at different time points or under different (pathologic) breathing conditions for the purpose of shedding new light on pulmonary physiology at microscopic resolutions. For the complete series of the three different pressure inflations, the single-projection entrance dose was 334 mGy adding up to a total absorbed dose of about 90 Gy for approximately 450 projections per scan. This is due to the fact, that not all X-ray photons are used for imaging, as they are partially absorbed in other anatomical features such as ribs. Although this value represents an acceptable upper dose limit for terminal (acute) experiments where no immediate effects are expected on the investigated lung tissue^32–36^, we did not attempt to further optimize the experimental setup in view of further reduction of the X-ray radiation dose.

From the imaging point of view, we note that the shortest scan time is determined by the heartbeat frequency and in our case was in the range of about 2 minutes per scan. High-quality images, i.e. those resolving structural features, were obtained at pixel sizes of 2.9 × 2.9 μm^2^ while further improvements in spatial resolution would necessitate shorter exposure times when imaging at pixel sizes of 1.1 × 1.1 μm^2^. In any case, we showed that the time point at which each tomographic projection is taken within the cardiac cycle is decisive for obtaining motionless images. As a first application we investigated the quasi-static inflation patterns at the alveolar scale. We discovered an intra- and inter-lobar distension pattern. However, due to the low number of samples this result has to be confirmed in additional experiments. At this scale, and within the investigated regions, the lungs can be described by heterogeneous up-scaling, meaning that alveolar structures heterogeneously increase in volume. Although our method will not replace low-resolution lung imaging studies where the whole thoracic field of view is required for investigating certain aspects, we believe that it will facilitate further research on the alveolar scale in high-resolution lung imaging. In particular, we showed that *in vivo* experimental conditions are preferred before drawing in-depth physiological conclusions from lung imaging experiments in a *post mortem* context.

## Methods

### Animal preparation

We used 9-days old newborn rats (*n* = 3, Wistar Bern, central animal facility of the University of Bern) for the experiments. All experiments were performed according to the Protection of Animal Act. They were approved and supervised by the veterinary authorities of the Cantons of Aargau and Bern. The detailed procedure in terms of animal preparation and anesthesia is summarized with the following three points:

1. **Intubation**: First, the animal is anesthetized with an injection of a mixture of fentanyl (Fentanyl-Janssen, Janssen-Cilag AG, Zug, Switzerland), midazolam (Dormicum, Roche Pharma AG, Reinach, Switzerland) and medetomidine (Domitor, Orion Corporation, Espoo, Finland), which takes about 20 minutes to put the animal into deep sleep, while still spontaneously breathing. Subsequently, the animal is intubated with a 24 gauge IV catheter by performing a tracheotomy and placed into an in-house designed sample-holder (Fig. 6a). The endotracheal cannula is fixed on the sample-holder (Fig. 6b) to remain rigid during the following tomographic image acquisition (sample rotation).

**Figure 6:**
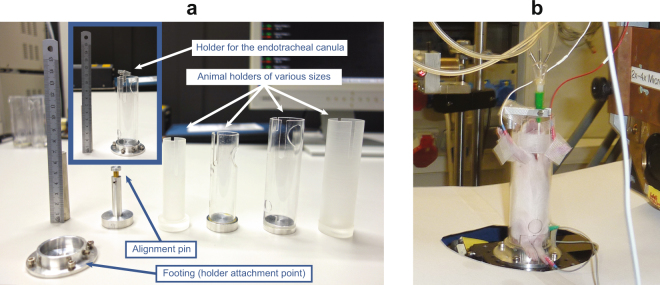
In-house designed animal sample holders (**a**) for different sizes of animals. An anesthetized 9 days old rat (**b**) is shown during image acquisition.
2. ***In vivo*** **imaging**: The animal (together with the sample-holder) is then transferred to the beamline hutch, mounted on the sample manipulator stage and ventilated through the endotracheal canula with a small animal’s ventilator (FlexiVent, SCIREQ Inc., Montreal, Canada) with an FiO_2_ of 0.5 containing 2–3% isoflurane (Baxter AG, Volketswil, Switzerland). By doing so, a standard ventilation pattern with a tidal volume of 10–20 ml/kg is applied at a frequency of 150 breaths per minute, which has been verified independently (n = 5) to yield physiological blood gas values (in terms of CO_2_ levels). This is significant since the setup at the beamline differs slightly from a standard “ventilation” setup: the animal remains in an upright position and the ventilation tubes are longer compared to the standard calibration of the small-animal ventilator. Prior to starting the image acquisition, the body temperature is measured and kept constant with a heating lamp (see below) during the experiment. At the same, heart rate is monitored continuously by putting small pins intracutaneously in the three extremities and connected to the ECG-device (SA Instruments, Inc., Stony Brook NY, USA). The ECG-electrodes are mounted in a way that they don’t interfere with the X-ray beam. Both, the cardiac activity and the animal’s ventilation are monitored continuously via a portable PC (personal computer). The desired region of interest (ROI) for imaging is aligned visually with an off-beam sample alignment setup^31^. During image acquisition, the animal is monitored from outside the beamline hutch. The explicit X-ray imaging procedure is explained below.
3. ***Post mortem*** **imaging**: After the *in vivo* image acquisition is finished, an overdose of pentobarbital (100–150 mg/kg) is administered to the animal. The animal is still being ventilated constantly and its heart activity monitored. In the 10 − 20 minutes following the pentobarbital injection the animal shows bradycardia until complete cardiac arrest. At this time point, the first *post mortem* images are then acquired.

With the described protocol it takes about 30 − 40 minutes from the first injection of anesthetic medicaments to the first acquired images. The whole imaging experiment requires approximately two hours per animal. As described in the following, the tomographic image acquisition was then directly triggered by inducing a breath-hold with a predefined inflation pressure (5, 10 and 15 cmH_2_O). After each breath-hold phase the animals were normally ventilated for about 10 min in order to recover from the long breath-holds.

### Image acquisition and reconstruction

**Figure 7:**
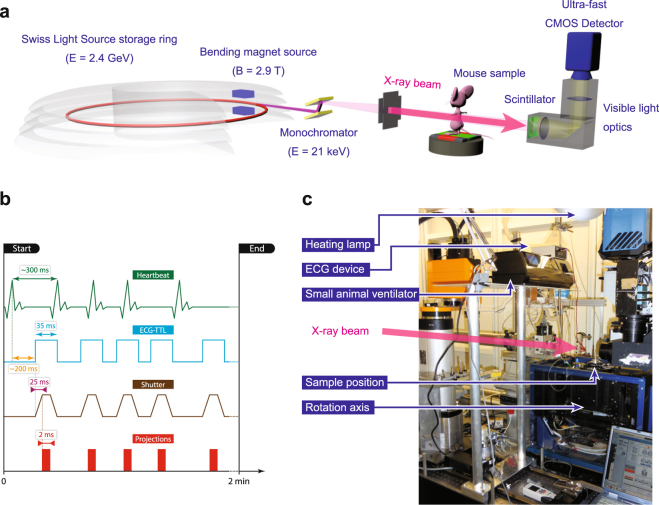
Schematic description of the imaging layout (**a**) at the X02DA TOMCAT beamline with the (**b**) complete triggering schematic for the prospective heartbeat-triggered gating technique and (**c**) the final *in vivo* endstation design with all accompanying components.

The realization of the heartbeat-gated *in vivo* tomographic imaging technique is based on a number of previous works. The experimental setup relies on our previously developed dose optimized imaging settings^40^ while the hardware implementation has been integrated into a flexible multi-purpose imaging endstation^31^. The complete imaging layout and acquisition scheme are depicted in Fig. 7. The experiment was carried out at the X02DA TOMCAT beamline (Fig. 7a) of the Swiss Light Source (SLS) at the Paul Scherrer Institute (Villigen, Switzerland), where the X-ray beam is produced by a 2.9 T bending magnet on a 2.4 GeV storage ring (with ring current *I* = 400 mA, top-up mode). A monochromatized (with a double-multilayer monochromator) X-ray energy of 21 keV is set in conjunction with a sample-to-source distance of 25 m. We used a high-speed CMOS detector (pco.Dimax) coupled to visible-light optics with a 150 μm and a 20 μm thick scintillator (LuAG:Ce) for the two different optics in use, yielding effective pixel sizes of 2.9 × 2.9 μm^2^ and 1.1 × 1.1 μm^2^. For these two optics, the fields of view were adjusted with horizontal and vertical slits, located just before the sample and producing beam sizes of 5.8 × 2.7 mm^2^ and 2.2 × 2.2 mm^2^, respectively. For the choice of the scintillating material and thickness it was crucial to optimize for efficiency, while allowing only a slight decrease in spatial resolution of the imaging system^41^. The sample to detector distance *z* was set to 100 mm, yielding an optimal trade-off between contrast-to-noise ratio and resolution settings^40^ and at the same time keeping the penumbral blurring at a minimum, i.e. with projected X-ray source sizes (full width at half maximum) being 0.51 μm and 0.18 μm in the horizontal and vertical direction, respectively^42^.

Our novel approach is based on prospective cardiac gating where each projection image had to be triggered by a heartbeat with an accurately adjusted delay. This original image acquisition protocol is depicted schematically (Fig. 7b) and explained as follows:

1. After the animal is placed onto the rotation stage, following the animal preparation procedure (see above), the ECG-signal is continuously monitored. From the ECG device a particular delay is set according to the heart rate of the investigated animal (e. g. 200 ms) after which the ECG-device fires TTL (transistor-transistor logic) signals of certain durations (e. g. 35 ms) after every heartbeat.
2. The TTL-signals directly trigger the X-ray shutter opening (and closing), with a delay of 25 ms due to its internal mechanics.
3. In parallel, every TTL-signal from the ECG also triggers the camera exposure. The camera, however, after receiving the TTL-signal acquires an image only after a pre-defined delay which is adjusted corresponding to the shutter opening delay (25 ms, as stated above).
4. Shortly before starting the scan, the total scan time *t*_scan_ is calculated in dependency on the actual heartbeat rate and the desired number of projections:
  1 $${t}_{{\rm{scan}}}[s]=\frac{60\,\times {n}_{{\rm{proj}}}}{{f}_{{\rm{HB}}}\,[\mathrm{BPM}]}$$
  where *n*_proj_ is the number of projections and *f*_HB_ is the heartbeat frequency given in “beats per minute”. Afterwards, the rotation speed is adjusted in accordance to the total scan time.
5. All the described triggering connections become effective and the actual image acquisition is started when a breath-hold phase is induced at a predefined inflation pressure, after which the rotation axis begins to rotate slowly with the adjusted rotation speed. During the subsequent constant 180°-rotation a tomographic image is then acquired each time the ECG detects a heartbeat.

With these settings the total scan time is in the range of 90 − 140 s with a total number of 400 − 450 projections, while the single-exposure times for each projection were set to 3 ms for the 2.9 μm-pixel-size and to 5 ms for the 1.1 μm-pixel-size optics, respectively. The associated entrance doses were 334 mGy and 557mGy for the 3 ms and 5 ms exposure times, respectively. Since during the breath-hold phases the animals usually exhibit very irregular heartbeats, the detector is caused to acquire images also very irregularly. As a consequence, the tomographic projections are not sampled in an equiangular way, which in the case of standard tomographic reconstructions would produce significant artifacts (Fig. 1b). This problem can be overcome, however, if the explicit angle of every tomographic projection is saved and utilized in the CT-reconstruction step (e. g. during back projection). For this reason, all trigger signals from all devices are recorded within a digital signal acquisition module (DAQ)^31^, which, after a simple post-processing procedure, allows for assigning every tomographic projection image to its explicit tomographic angle. After doing so, the tomographic projections are then phase-retrieved^18^, after having empirically selected the phase retrieval parameters (delta and beta) by visual inspection analysis of the reconstructed datasets. Finally, the phase-retrieved projections are CT reconstructed with the *gridrec* algorithm by using the recorded angles^43^.

### Data Availability

The datasets generated during and/or analysed during the current study are available from the corresponding author on reasonable request.
